# Book and Pamphlet Notices

**Published:** 1859-07

**Authors:** 


### BOOK AND PAMPHLET NOTICES.

A Manual of Elementary Chemistry, Theoretical and Practical. By George Fownes, F.R.S. From the seventh revised and corrected London edition. Edited by Robert Bridges, M.D., Professor of Chemistry in the Philadelphia College of Pharmacy, etc. Philadelphia: Blanchard & Lea. 1859. From W. B. Keen.

"Fownes' Manual" has already been adopted as a text book in many of the medical colleges in this country, which shows sufficiently the estimation in which it is held. This from the seventh London edition is fully corrected, so as to represent the present state of chemical science, and render the work deserving of the same rank which it has hitherto maintained.

---

Woman: her Diseases and Remedies. A Series of Letters to his Class. By Charles D. Meigs, M.D., Professor of Midwifery and the Diseases of Women and Children, in the Jefferson Medical College at Philadelphia; Member of the American Medical Association, of the American Philosophical Society, and the Council; late Vice-President of the College of Physicians of Philadelphia; of the Swedish Society of Physicians at Stockholm; late one of the physicians to the lying-in department of the Pennsylvania Hospital, etc. etc. Fourth edition, revised and enlarged. Philadelphia: Blanchard & Lea. 1859. From W. B. Keen.

In all, except in one particular, this book presents internal evidence of being the work of a veteran in the profession, for in the extended experience from which the author draws his teachings, and the graceful authority with which he enforces them, we could not fail to recognize the hand of a master. But, from the peculiarly free, easy and florid style which pervades the entire volume, so entirely different from all that we are accustomed to meet in standard works upon the science or art of medicine, we would be led to the belief, that it must be the work of one who is still inhaling the atmosphere of vernal hope and buoyancy, rather than of one who, as the author says of himself in another place, "is walking in the descending path of life."

We greet this new edition of Dr. Meigs' work on Woman with much pleasure, and commend it to the profession, especially to the younger members, who may receive much valuable instruction from its pages, conveyed in a pleasing style. The teaching throughout the work reflects the highest credit upon the head and heart of the author, as does the mechanical execution of the book upon the skill of its enterprising publishers.

M.

---

IMPORTANCE OF THE STUDY OF LEGAL MEDICINE; a Lecture introductory to the Course on Medical Jurisprudence, at the New York Medical College. By JAMES WYNNE, M.D., Lecturer on Medical Jurisprudence in the New York Medical College.

From this instructive lecture we extract a passage on the subject of analytical chemistry, which contains a well deserved tribute to several of our friends, including one of our colleagues in Rush Medical College. The lecture will well repay a careful perusal:

"In this class of cases it is always important to obtain the opinion of experts, whose studies and facilities for analysis give great and deserved weight to their opinions. The science of analytical chemistry is here of chief importance, and such men as Booth, of Philadelphia, Piggott, of Baltimore, J. Lawrence Smith, of Louisville, Blaney, of Chicago, and Campbell, Morfit, and Doremus, of this city, whose lives are devoted to this pursuit, and in which they have obtained deserved reputation, cannot be too widely known."

